# Autocrine signalling as cause of sensitized cAMP formation

**DOI:** 10.1186/1471-2210-11-S2-A37

**Published:** 2011-09-05

**Authors:** Christian Nanoff, Qiong Yang, Edin Ibrišimović

**Affiliations:** 1 Institute of Pharmacology, Center of Physiology and Pharmacology, Medical University of Vienna, 1090 Vienna, Austria

## Background

Cyclic adenosine monophosphate (cAMP) is a second messenger essential for neural functions. The propensity of a nerve cell to mount a cAMP response may be conditional, e.g. depending on a preceding exposure to stimuli (as in long term potentiation).

## Methods

We measured cAMP formation in SH-SY5Y neuroblastoma cells at various stages of differentiation to a neuronal phenotype. We analyzed expression pattern of enzymes involved in regulated cAMP production, differentiation markers and protein composition of culture supernatant.

## Results

SH-SY5Y cells (a model nerve cell) required differentiation to produce cAMP in substantial amounts; in undifferentiated proliferating cells, forskolin or activation of G_s_-coupled receptors barely stimulated cAMP formation. A cell-autonomous process induced sensitization. The process relied on an autocrine factor, which we identified as Dickkopf1 protein. Serum protein quenched the activity of Dickkopf1; conversely, serum deprivation allowed for sensitization to unfold. The effect of Dickkopf1 was mediated by a high-affinity receptor activated at concentrations of ≤1 nM. In accordance with its cognate function as Wnt antagonist, sensitization was a consequence of suppressing the canonical Wnt signaling pathway; the inhibitors of glycogen-synthase kinase-3β, lithium chloride and, in addition, valproic acid mimicked Wnt signals and diminished the extent of sensitized cAMP formation. We found that in differentiated cells, expression of the α-subunit of G_s_ (Gα_s_) increased due to activation of the GNAS gene. Although sufficient to support G_s_-coupling of the A_2A_ adenosine receptor, increased Gα_s_ alone failed to enhance receptor-stimulated cAMP formation. We infer that sensitized cAMP formation reflected increased responsiveness of the catalyst, adenylyl cyclase, to stimuli.

## Conclusions

SH-SY5Y provide for a nerve cell model to study the effect of Wnt signaling on regulated cAMP formation. Our data suggest that mood stabilizing agents act by reducing the ability of nerve cells to produce cAMP.

